# Probiotic Lactobacilli Isolated from Kefir Promote Down-Regulation of Inflammatory Lamina Propria T Cells from Patients with Active IBD

**DOI:** 10.3389/fphar.2021.658026

**Published:** 2021-04-15

**Authors:** Renata Curciarello, Karina E. Canziani, Ileana Salto, Emanuel Barbiera Romero, Andrés Rocca, Ivan Doldan, Emmanuel Peton, Santiago Brayer, Alicia M. Sambuelli, Silvina Goncalves, Pablo Tirado, Gustavo J. Correa, Martín Yantorno, Laura Garbi, Guillermo H. Docena, María de los Ángeles Serradell, Cecilia I. Muglia

**Affiliations:** ^1^ Instituto de Estudios Inmunológicos y Fisiopatológicos (IIFP), CONICET-Departamento de Ciencias Biológicas, Facultad de Ciencias Exactas, Universidad Nacional de La Plata, Asociado CIC PBA, La Plata, Argentina; ^2^ Unidad Endoscopía, Hospital de Gastroenterología Dr. Carlos Bonorino Udaondo, Ciudad Autónoma de Buenos Aires, Argentina; ^3^ Unidad de Proctología, Departamento de Cirugía, Hospital de Gastroenterología Dr. Carlos Bonorino Udaondo, Ciudad Autónoma de Buenos Aires, Argentina; ^4^ Sección de Enfermedades Inflamatorias Del Intestino, Hospital de Gastroenterología Dr. Carlos Bonorino Udaondo, Ciudad Autónoma de Buenos Aires, Argentina; ^5^ Área de Enfermedad Inflamatoria Intestinal, Sala de Endoscopía, Servicio de Gastroenterología, Hospital Interzonal General de Agudos General San Martín, La Plata, Argentina; ^6^ Cátedra de Microbiología, Departamento de Ciencias Biológicas, Facultad de Ciencias Exactas, Universidad Nacional de La Plata, La Plata, Argentina

**Keywords:** ulcerative colitis, Crohn’s disease, probiotics, immunomodulation, mucosal samples, *Lactobacillus kefiri* CIDCA 8348

## Abstract

Ulcerative colitis and Crohn’s disease, the two main forms of inflammatory bowel disease (IBD), are immunologically mediated disorders. Several therapies are focused on activated T cells as key targets. Although *Lactobacillus kefiri* has shown anti-inflammatory effects in animal models, few studies were done using human mucosal T cells. The aim of this work was to investigate the immunomodulatory effects of this bacterium on intestinal T cells from patients with active IBD. Mucosal biopsies and surgical samples from IBD adult patients (*n* = 19) or healthy donors (HC; *n* = 5) were used. Lamina propria mononuclear cells were isolated by enzymatic tissue digestion, and entero-adhesive *Escherichia coli*-specific lamina propria T cells (LPTC) were expanded. The immunomodulatory properties of *L. kefiri* CIDCA 8348 strain were evaluated on biopsies and on anti-CD3/CD28-activated LPTC. Secreted cytokines were quantified by ELISA, and cell proliferation and viability were assessed by flow cytometry. We found that *L. kefiri* reduced spontaneous release of IL-6 and IL-8 from inflamed biopsies *ex vivo.* Activated LPTC from IBD patients showed low proliferative rates and reduced secretion of TNF-α, IL-6, IFN-γ and IL-13 in the presence of *L. kefiri*. In addition, *L. kefiri* induced an increased frequency of CD4^+^FOXP3^+^ LPTC along with high levels of IL-10. This is the first report showing an immunomodulatory effect of *L. kefiri* CIDCA 8348 on human intestinal cells from IBD patients. Understanding the mechanisms of interaction between probiotics and immune mucosal cells may open new avenues for treatment and prevention of IBD.

## Introduction

Inflammatory bowel disease (IBD) comprises a complex group of chronic relapsing diseases among which the most conspicuous are ulcerative colitis (UC) and Crohn's disease (CD). Patients with IBD suffer chronic inflammation of the bowel mucosa that may affect the mucosal layer (UC) or the whole bowel wall (CD) (Torres et al., 2017; Ungaro et al., 2017). The etiology of these diseases is largely unknown, although factors such as diet, certain genes related to the sensing of luminal microbes, secretion of antimicrobial peptides and autophagy have shown to be associated (J. S. Lee et al., 2021; Kaser and Blumberg 2011). Changes in microbiota composition are typically observed in IBD patients. These findings, along with results obtained from animal models, including the fact that germ free mice do not develop experimental colitis, highlight the impact of the microbiota composition in the pathogenesis of these disorders (Onderdonk et al., 1977).

Metagenomic strategies have revealed an altered gut microbial composition in IBD patients compared to healthy subjects, known as dysbiosis (Liu et al., 2020). IBD patients usually present a reduced bacterial diversity, with low levels of Firmicutes and Bacteroidetes and increased levels of facultative anaerobic Proteobacteria and Bacilli. However, the implication of these findings for pathogenesis is not clear. Also, persistent bacterial infection by enteric bacteria, such as adherent-invasive *Escherichia coli*, has been observed (Abdelhalim et al., 2020; J. G. Lee et al., 2019). Intensive research is being carried out to determine whether changes in microbiota are causative or a consequence of the chronic inflammation observed in IBD. Inflammation probably comes from a sum of effects: an increased amount of mucosal associated bacteria, along with high intestinal permeability also present in these patients suggest that bacteria could penetrate the epithelial barrier, thus contributing to inflammation. These microorganisms in turn may promote the release of pro-inflammatory factors such as TNF-α, which boost inflammation (Shawki and McCole, 2017). Altered *trans*-cellular and *para*-cellular permeability have been described in IBD, evidenced by the presence of intracellular bacteria inside epithelial cells, and by modified tight junction protein expression and increased myosin light chain kinase (MLCK) activity (Yu Chia-Hui, 2018). Moreover, gut permeability can be influenced by changes in metabolites produced by the microbiota (Schlegel et al., 2020; J. S. Lee et al., 2021). The healthy gut microbiome produces bioactive metabolites, including short chain fatty acids (SCFA), which contribute to intestinal homeostasis and epithelial cell nutrition (Postler et al., 2017). Diminished levels of these molecules could favor an impaired barrier function and inflammatory environment.

The role of CD4^+^ T lymphocytes is critical in IBD. In CD, IL-12 signaling induces the differentiation of CD4^+^ T cells into IFN-γ secreting cells, while IL-23 contributes to the differentiation of CD4^+^ T cells into Th1 and Th17, thus increasing IFN-γ and IL-17 secretion, respectively. IL-6, IL-23 and TGF-β also participate in CD pathogenesis. In UC, CD4^+^ T lymphocytes also secrete IL-4 and IL-13, which contribute to tissue damage (Zenewicz et al., 2009). In addition, activated CD4^+^ T cells have increased proliferation rate and are resistant to apoptosis in these pathologies (Schmitt et al., 2019). Anti-TNF-α therapies target these activated T cells, inducing T cell apoptosis, but unfortunately, many patients do not respond or become refractory within years of treatment, and require surgery (Yanai and Hanauer, 2011). Consequently, great effort is being made to develop new therapies for IBD, aimed to modulate T cell response.

Probiotics were defined several years ago as “live microorganisms that confer a health benefit to the host when administered in adequate amounts” (Food and Agriculture Organization and of the United Nations/World Health Organization, 2002). Recently, the term “postbiotic” has come to be used and refers to the functional bioactive compounds generated during microbial fermentation processes, including extracellular polysaccharides, (SCFA) and different microbial cell components, which can have beneficial effects on host health (Wegh et al., 2019). Probiotics have been studied as having beneficial properties in murine and rat models of colitis. Different strains of *Bifidobacterium*, *E. coli* and *Lactobacillus* have shown anti-inflammatory effects (Jakubczyk et al., 2020). Even though data from probiotics treatment in patients are controversial, evidence of their usefulness in combination with pharmacological treatments is arising (Shanahan and Quigley, 2014; Qiao et al., 2016; Fan et al., 2019; Ahn et al., 2020). Nevertheless, basic studies regarding their effect on IBD lamina propria cells are scarce. Kefir is an ancient product traditionally obtained by fermentation of milk with kefir grains, and many health-promoting properties have been associated with its consumption (Farag et al., 2020). Kefir grains are composed of different species of bacteria and yeasts that live symbiotically in a complex matrix constituted by proteins and polysaccharides (Bengoa et al., 2019). *Lactobacillus kefiri* is one of the most important lactobacilli retrieved from kefir, with reported quantification of around 10^8^ bacteria/mL in fermented milk (Garrote et al., 2005). Most *L. kefiri* strains are safe for consumption and resistant to the harsh conditions of the gastrointestinal tract. Moreover, different beneficial effects, including immunomodulation and prevention of metabolic disorders, have been reported for these species (Slattery et al., 2019). In particular, the kefir-isolated strain *L. kefiri* CIDCA 8348 has shown to be sensitive to several antibiotics, to lack virulence factors, and to be safe for oral consumption in mice (Carasi et al., 2014). Noteworthy, mice orally treated with this strain showed reduced expression of pro-inflammatory molecules and an up-regulation of anti-inflammatory mediators, as well as secretory IgA and mucins in the gut (Carasi et al., 2015). Moreover, it was reported that administration of *L. kefiri* CIDCA 8348 prevents the deleterious effects of a fructose-rich diet in a murine model, exerting an anti-inflammatory activity in the adipose tissue (Zubiría et al., 2017).

In this work, we studied the immunomodulatory properties of *L. kefiri* CIDCA 8348 on CD4^+^ T lymphocytes from the lamina propria of IBD patients. This *Lactobacillus* strain diminishes the proliferation of these cells and the secretion of pro-inflammatory cytokines through an NF-κB dependent pathway.

## Materials and Methods

### Patients and Tissue

Surgical samples of colon or rectum from patients who underwent partial or total colectomy, and endoscopic colonic biopsies were taken from macroscopically inflamed mucosa of IBD adult patients affected by CD (*n* = 8) or UC (*n* = 11). The diagnosis was made according to clinical, endoscopic and histological criteria. The extent and location of the UC and CD were evaluated during colonoscopy. Clinical activity in UC was evaluated by total Mayo score (inactive ≤2, mild activity three to five, moderate seven to nine, severe 10–12) and by Harvey-Bradshaw Index (Inactive score <5; mild activity ≥5, moderate ≥7, severe ≥16) in CD (Table 1) (Peyrin-Biroulet et al., 2016). In addition, mucosal samples were collected endoscopically from the colon of adult subjects who were neither diagnosed with IBD nor any other inflammatory condition of the gut. Samples of healthy mucosa were obtained from surgical specimens of colorectal cancer partial colectomies. These specimens constituted the "healthy control patient" samples (HC, *n* = 5). The local Ethics Committee (*Comité de Ética en Investigaciones, Hospital de Gastroenterología Carlo B. Udaondo, Ciudad Autónoma de Buenos Aires,* Res. 07–07–2016) approved the protocols and informed written consent was obtained from every patient.

**TABLE 1:** Clinical features of healthy control (HC) and IBD patient groups.

	HC group	IBD group	
		UC	CD
Number of patients	5	11	8
Sex (*n* = ***patients***)	Female = 3, Male = 2	Female = 6, Male = 5	Female = 7, Male = 1
Age of the patients [average (range)]	51 (23–73) y	39 (18–64) y	38 (21–59) y
Site of sampling (*n= patients*)^a^	Left colon (2) rectum (3)	Cecum (1), right (2), left (4), transverse (2), sigmoid (3), rectum (8)	Cecum (2), right (3), left (2), transverse (1), rectum (2)
Endoscopic activity at sampling time (*n= patients*)	No activity = 5	mild = 6, moderate = 3 severe = 2	No activity = 1^b^, mild = 1, moderate = 4 severe = 2
Treatment at sampling time (*n= patients*)^c^	Not applicable	Adalimumab (1) corticosteroids (3) mesalazine (9) azatioprine (4)	Adalimumab (2) infliximab (1) corticosteroids (4) mesalazine (3) azatioprine (2)

UC = ulcerative colitis, CD = Crohn's disease, age is expressed in years (y).

^a^ Samples were taken from more than one site in some patients.

^b^ *Uninflamed samples were only used for LPTC in vitro assays*.

^c^ Some patients were under multiple pharmacological treatment.

### Bacteria and Conditioned Medium

*Lactobacillus kefiri* CIDCA 8348 belonging to the collection of the Centro de Investigación y Desarrollo en Criotecnología de Alimentos (CIDCA, CONICET-UNLP-CIC, Argentina) was used. The strain was cultured in deMan-Rogosa-Sharpe (MRS) broth (Difco, Beauvais, France) at 37°C for 48 h in aerobic conditions. Bacteria were harvested, washed twice and finally resuspended in sterile phosphate-buffered saline (PBS) at OD^550^ = 2.0 (approx. 1–2x10^8^ cfu/ml). In order to prepare the *L. kefiri*-conditioned medium (CM), bacterial suspension at OD^550^ = 0.1 in Ultraculture medium was incubated for 24 h at 37°C and 5% CO_2_. Then, bacteria were removed by centrifugation and supernatant was collected and stored at −20°C until used. Entero-adhesive (EA) *E. coli* was grown in Luria Bertani Broth to OD^550^ = 0.8. Cells were then harvested by centrifugation and resuspended in sterile PBS. Extracts were sonicated for 10 pulses at 100% and centrifuged al 10.000×*g* for 15 min. Protein concentration of the resulting solution was assayed by bicinchoninic acid assay (Pierce, Thermo Fisher Scientific, Rockford, IL, United States) and stored at −20°C. Prior to use, extracts were thawed and diluted in fresh medium to the desired concentration.

### Organ Cultures

Endoscopic mucosal biopsies from control subjects (*n* = 5) or IBD patients (*n* = 13) were placed (one biopsy per well) in 24-well plates. Individual biopsies were cultured in 300 μL of serum-free RPMI 1640 medium (Gibco, Thermo Fisher Scientific, Rockford, IL, United States), supplemented with 100 U/mL penicillin and 100 μg/ml streptomycin, and cultured at 37°C, 5% CO_2_, with or without probiotics and/or 10 ng/ml TNF-α. After 24 h of *ex vivo* culture, supernatants of mucosal biopsies were collected and stored at −70°C until used.

### Lamina Propria Mononuclear Cell Isolation

The mucosa layer of surgical pieces was mechanically separated from the full-thickness surgical specimen. The epithelial layer was removed with 1 mmol/L ethylenediaminetetraacetic acid (EDTA) and 1 mmol/L dithiothreitol (DTT) in 1 mM HBSS (Gibco, Thermo Fisher Scientific, Rockford, IL, United States). After stirring for 1 h at 37°C, the supernatant was removed. The remaining tissue was minced with a scalpel and digested with type 1 A collagenase (1 mg/ml; Sigma-Aldrich, St. Louis, MO, United States) and DNAse (10 IU/ml, Roche, Thermo Fisher Scientific, Rockford, IL, United States) in RPMI-1640 medium containing 10% fetal bovine serum (FBS), 100 U/mL penicillin, and 100 μg/ml streptomycin for 2 h with stirring at 37°C and 5% CO_2_. Biopsies were washed three times in HBSS containing EDTA and DTT with stirring and then digested as described above. Cells were filtered through 40 μm cell-strainers (Becton Dickinson, Franklin Lakes, NJ, United States) and washed with RPMI-1640 medium containing 10% FBS, 100 U/mL penicillin, and 100 μg/ml streptomycin.

### Generation of Lamina Propria T-Cell Lines (LPTC)

LPMC were washed and cultured in 300 µL of serum-free Ultraculture medium (LONZA, Basel, Switzerland), supplemented with 2 mM glutamine, 20 μM 2-mercaptoethanol, and Antibiotic-Antimicotic (Gibco, Thermo Fisher Scientific, Rockford, IL, United States). In order to obtain specific LPTC, cells were stimulated with entero-adhesive *E. coli* extracts (0.5 μg/ml) at 37°C, 5% CO_2_. Cultures without *E. coli* extract served as controls. Four days later, cells were treated with recombinant human (rh) IL-2 (10 U/mL, Preprotech, Rocky Hill, NJ, United States), rhIL-7 (10 ng/ml, Preprotech, NJ, United States) and rhIL-15 (10 ng/ml, Preprotech, Rocky Hill, NJ, United States) as reported by Rabinowitz et al. (2013). After 5 days, viable T cell blasts were enriched by Ficoll-Paque ™ (GE, Healthcare, Life Sciences, Danderyd, Sweden) gradient and then incubated in 96-well round-bottom cultures plates, in Ultraculture medium, supplemented with the cytokines mentioned above, 10% human AB^+^ plasma and irradiated peripheral blood mononuclear cells PBMC (1 × 10^5^) (Bohle et al., 2003). Cells were expanded in this same medium twice a week until enough cells were obtained, thus generating EA *E. coli* specific LPTC (from now on LPTC).

### LPTC Cultures and Proliferation Assays

After expansion, LPTC were rested without further feeding for 10 days. Cells were washed, labeled with CFSE (Sigma-Aldrich, St. Louis, MO, United States) proliferation dye and stimulated with human anti-CD3 and anti-CD28 antibodies (1 μg/ml, eBioscience, San Diego, CA, United States), *L. kefiri* (2:1 bacteria:eukaryotic cell relation), *L. kefiri* conditioned media or combinations thereof. EA *E. coli* cultures were used as a positive control, also in a 2:1 relation to lymphocytes. Negative controls without the addition of stimuli were included. Assays were performed in serum-free AIMV medium (Thermo Fisher Scientific, Rockford, IL, United States). After 4 days, culture supernatants were harvested for cytokine evaluation and cells were stained with anti-CD4-APC (BD Pharmingen, San Diego, CA, United States), 7-AAD (BD Pharmingen, San Diego, CA, United States) and flow cytometry was performed using a FACS CALIBUR (BD, Franklin Lakes, NJ, United States). Flow cytometry data from two independent experiments from each patient was analyzed using FlowJo software (BD, Franklin Lakes, NJ, United States).

For intracellular FOXP3 staining, cells were stimulated as previously indicated. After 4 days cells were harvested and stained with anti-CD4-APC (BD Pharmingen, San Diego, CA, United States). Cells were then treated with BD Cytofix/Cytoperm Fixation/Permeablization Kit (BD Pharmingen, San Diego, CA, United States) and stained with anti-FOXP3-PE (BD Pharmingen, San Diego, CA, United States). Events were acquired with a FACS CALIBUR (BD, Franklin Lakes, NJ, United States). Lymphocytes were gated in the FSC/SSC scatter plot. CD4^+^ lymphocytes were then selected from this gate and analyzed for FOXP3 staining. The negative threshold was set using fluorescence minus one controls (FMO). Duplicate independent experiments were performed for each patient.

### ELISA

Cytokines were quantified in organ culture and LPTC supernatants (each sample was tested in duplicate), following manufacturer's instructions: human IL-6 (R&D systems, Minneapolis, MN, United States), human IL-8 (BD, Franklin Lakes, NJ, United States), human IFN-γ and human TNF-α (ImmunoTools, Friesoythe, Germany), human IL-13 (Invitrogen, Thermo Fisher Scientific, Rockford, IL, United States), human IL-10 (R&D systems, Minneapolis, MN, United States), human IL-1β and human IL-17 A (Biolegend, San Diego, CA, United States).

### Western Blot

Protein extracts from LPTC incubated with anti-CD3/anti-CD28 alone or combined with *L. kefiri* for 30 min were used. Briefly, cells were harvested and lyzed with RIPA buffer (Sigma-Aldrich, St. Louis, MO, United States) in the presence of a protease inhibitor mixture (Sigma-Aldrich, St. Louis, MO, United States). Protein content was quantified by bicinchoninic acid assay (Pierce, Thermo Fisher Scientific, Rockford, IL, United States) and extracts were stored at –80°C until use. Protein samples were resolved on 10% SDS-PAGE under reducing conditions (BioRad Mini-Protean III; BioRad, Hercules, CA, United States), and transferred onto a nitrocellulose membrane for 1 h at 300 mA. Blots were blocked and probed with a rabbit anti-p65 antibody (Santa Cruz Biotechnology, Santa Cruz, CA, United States), followed by the appropriate HRP-conjugated secondary antibody (BioRad, Hercules, CA, United States). Protein bands were visualized by enhanced chemiluminescence (ECL Plus; GE Healthcare, Danderyd, Sweden) according to the manufacturer’s instructions. Blots were stripped and incubated with a rabbit anti-human *β*-actin antibody (Abcam, Cambridge, MA, United States) diluted 1:3,000, as an internal loading control. The bands were scanned with C digit scanner (LI-COR Biosciences, Lincoln, Nebraska, United States) and quantified using ImageJ software.

### Statistical Analysis

Statistical analysis was carried out using GraphPad Prism eight software (GraphPad Software, San Diego, CA, United States). The significance of the difference was determined using an independent-sample *t*-test or 1-way ANOVA after visual inspections of distribution using Q–Q plots and Shapiro–Wilk normality test analysis. In cases when data did not adjust to normal distribution, Wilcoxon matched paired test or Friedman statistics were applied. A *p*-value < 0.05 was considered statistically significant.

### Ethics Statement

The studies involving human participants were reviewed and approved by Ethics Committee of the Hospital de Gastroenterología Carlos B. Udaondo. The patients/participants provided their written informed consent to participate in this study.

## Results

### *Lactobacillus Kefiri* Reduces the Pro-Inflammatory Cytokine Secretion in Inflamed Biopsies from IBD Patients

**FIGURE 1:**
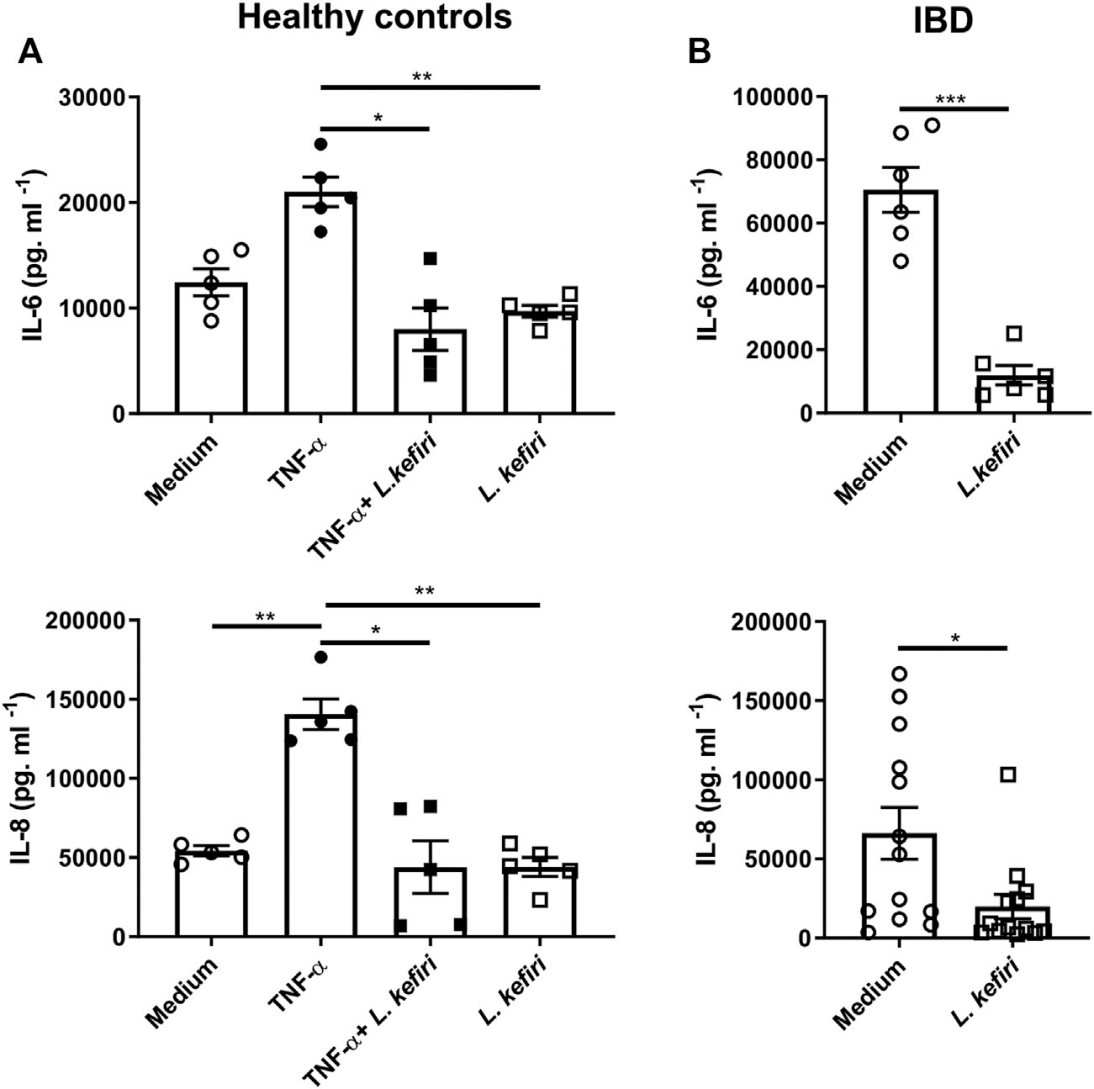
*Lactobacillus kefiri* modulates the secretion of IL-6 and IL-8 in organ culture **(A).** Biopsies from healthy donors (*n* = 5) were incubated o. n. with TNF-α, *L. kefiri* or a combination of both **(B)**. Biopsies form IBD patients (*n* = 13) were incubated with or without *L. kefiri.* Supernatants were collected and cytokines were assessed by ELISA. *p < 0.05, **p < 0.01, ***p < 0.001.

In order to evaluate the immunomodulatory effects of *L. kefiri* CIDCA 8348, we assayed IL-6 and IL-8 in culture supernatants of tissue explants from healthy subjects (*n* = 5) incubated with the pro-inflammatory stimulus TNF-α, with or without probiotics (Figure 1A). TNF-α promoted the secretion of IL-8 (*p* < 0.01), and a trend in IL-6 secretion (*p* = 0.0616) with respect to unstimulated healthy tissue. Explants exposed to *L. kefiri* produced reduced levels of these TNF-α -induced cytokines (*p* < 0.05). We then studied the effect of the bacteria on biopsies from inflamed mucosa of IBD patients (*n* = 13). *L. kefiri* significantly dampened the spontaneous release of pro-inflammatory cytokines *ex vivo* (*p* < 0.001) for IL-6 and IL-8 (*p* < 0.05) (Figure 1B). A significant similar suppression of IL-1β and IL-17 A secretion was observed in IBD samples (*p* < 0.01) (Supplementary Figure S1).

### *Lactobacillus Kefiri* Modulates the Cell Proliferation of Stimulated Microbiota-Specific Lamina Propria T Lymphocytes

**FIGURE 2:**
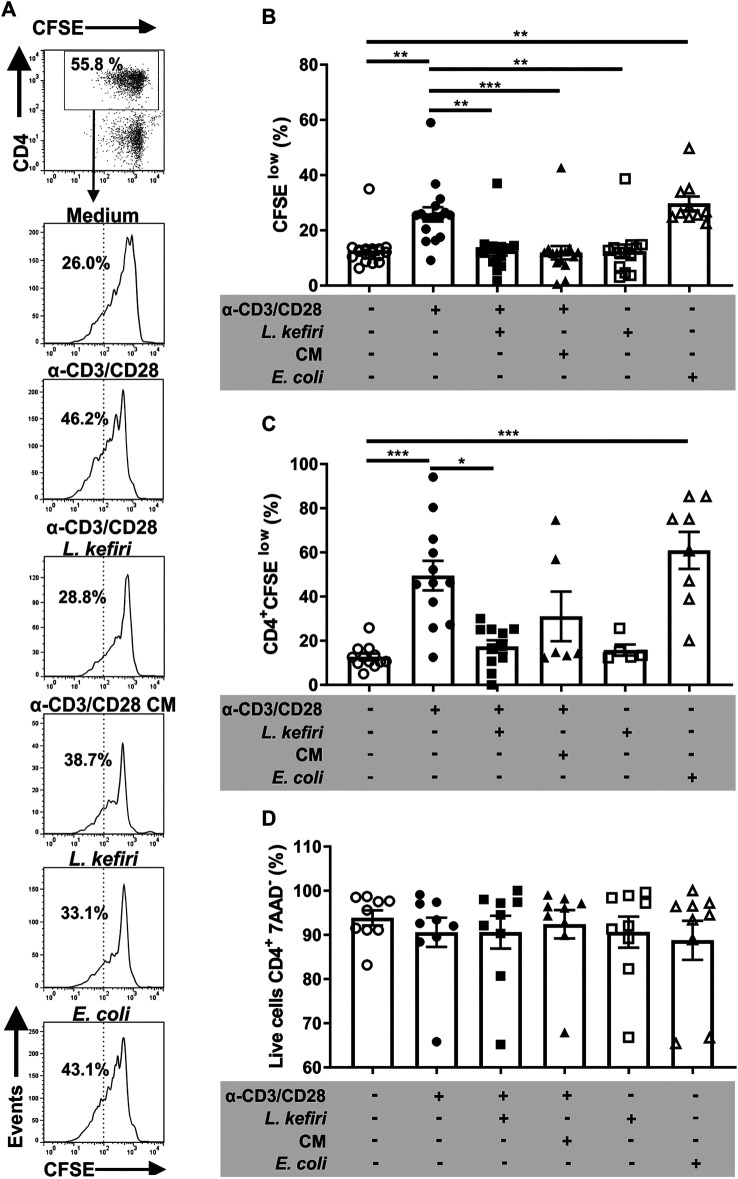
Proliferation and LPTC response are impaired by *L. kefiri* **(A).** LPTC were activated with anti-CD3 and anti-CD28 and incubated with live probiotic, conditioned medium (CM), EA ***E*** *. coli* or medium. Cell proliferation was assessed by flow cytometry with CFSE. The gating strategy is shown: CD4^+^ cells were gated from the lymphocyte gate on the forward and side scatter plot. Histograms for CFSE staining are shown separately for each stimulus. Representative results from one patient are shown **(B)**. LPTC proliferation shown as percentage of “CFSE low” staining population under each stimulus *in vitro.* Each symbol represents one independent experiment **(C)**. Proliferation of CD4^+^ LPTC is shown as percentage of CFSE low cells obtained by the strategy shown in A, each symbol represents one independent experiment **(D)**. CD4^+^ LPTC cell death was assessed by flow cytometry with 7-AAD staining, in the same LPTC cultures. *p < 0.05, **p < 0.01, ***p < 0.001.

Since EA *E. coli* is overrepresented in the microbiota of IBD patients, we expanded *E coli*-specific LPTC. We could not retrieve LPTC from HC since these cells did not survive long *in vitro* under EA *E. coli* extract stimulation. We therefore proceeded to generate LPTC from 17 colon samples from patients with active IBD. We found that EA *E. coli* extracts significantly increased (*p* < 0.01) the proliferation of LPTC from IBD patients (Figure 2A).

Aiming to study whether *L. kefiri* modulates IBD LPTC, anti-CD3/CD28-stimulated cells were co-incubated with the probiotics. We found that the increased proliferation of LPTC (*p* < 0.01) and CD4^+^ LPTC (*p* < 0.001) (Figures 2A–C) were significantly inhibited by incubation with *L. kefiri* (*p* < 0.01 and *p* < 0.05, respectively). Also, the cell viability remained unchanged in all conditions, as shown with 7AAD staining (Figure 2D). *E. coli* used as control promoted cell proliferation, thus confirming the specificity of T cells (*p* < 0.01 and *p* < 0.001, respectively).

Considering that lactic acid bacteria secrete SCFA with immunomodulatory properties, we also incubated LPTC with conditioned medium from *L. kefiri*. As depicted, we found that LPTC proliferation was significantly suppressed (*p* < 0.001), whereas for activated-CD4^+^ T cells, it did not reach statistical significance (*p* = 0.106) (Figures 2A–D).

### *Lactobacillus Kefiri* Ameliorates the Pro-Inflammatory Cytokine Secretion by Stimulated-Microbiota-Specific Lamina Propria T Cells

**FIGURE 3:**
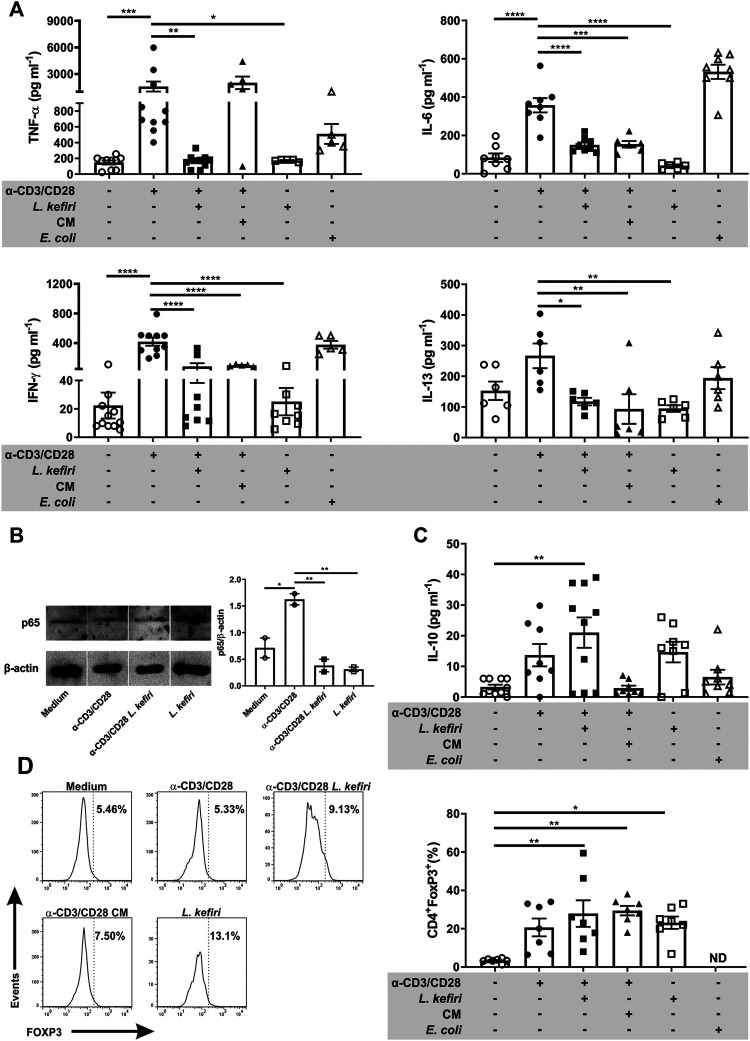
*Lactobacillus kefiri* modulates LPTC cytokine secretion, FOXP3 expression and NF-κB signaling **(A).** Effect of *L. kefiri* and CM on cytokine secretion from activated LPTC. Cytokines were assessed by ELISA **(B)**. Immunoblots of LPTC protein extracts after 30 min of stimulation (representative of two independent assays) and statistical analysis of intensities of bands corresponding to p65 **(C)**. Quantification of IL-10 by ELISA in supernatants of the same LPTC assays shown in **A (D).** Representative histograms of the flow cytometry data analysis of LPTC. Frequency of CD4^+^FOXP3^+^ T cells after 4 days incubation with live probiotic or CM. FOXP3^+^ cells were evaluated in CD4^+^ cells from the lymphocytes gate. Anti-CD3 and anti-CD28 antibodies were used for T cell activation. *p < 0.05, **p < 0.01, ***p < 0.001, ****p < 0.0001.

To further characterize the cellular response of these cells *in vitro*, we evaluated the secretion of pro-inflammatory cytokines by activated LPTC when co-incubated with *L. kefiri*. The stimulation of LPTC with anti-CD3/CD28 induced the secretion of TNF-α, IFN-γ, IL-6 and IL-13. The co-incubation of activated LPTC with the probiotic significantly diminished the secretion of these cytokines (Figure 3A, *p* < 0.01, *p* < 0.0001, *p* < 0.0001 and *p* < 0.05 respectively). Also, the incubation of the stimulated cells with CM significantly reduced the levels of the pro-inflammatory cytokines IFN-γ (*p* < 0.0001), IL-6 (*p* < 0.001) and IL-13 (*p* < 0.01).

We then investigated the activation of the NF-κB pathway on LPTC from patients with active IBD in the different culture conditions. We found that p65 levels were diminished in activated cells after a 30 min exposure to *L. kefiri* (Figure 3B, *p* < 0.01). *L. kefiri per se* did not trigger the NF-κB pathway.

Next, we analyzed the secretion of the tolerogenic cytokine IL-10 and we found it significantly increased in the supernatant of stimulated LPTC that were exposed to *L. kefiri* (Figure 3C, *p* < 0.01). To get a further insight into this suppressive effect, FOXP3 expression was also evaluated in LPTC by flow cytometry (Figure 3D). We found that this transcription factor was specifically induced in CD4^+^ LPTC by *L*. *kefiri*, irrespective of their activation with anti-CD3/anti-CD28 (*p* < 0.05). We also found that CM induced FOXP3 expression in activated LPTC (*p* < 0.01) (Figure 3D).

## Discussion

In IBD, dysregulated immune responses take place against the intestinal microbiota in genetically predisposed hosts, and several T cell subsets have been described to be involved in homeostasis breakdown. In this context, microbiota-specific T cells were identified in IBD patients and in animal models (Hepworth et al., 2015; Sorini et al., 2018). Intestinal T cells are target of several therapeutic procedures to constrain inflammation.

In our study, we aimed to evaluate the immunomodulatory properties of a probiotic *Lactobacillus* strain isolated from kefir on lamina propria CD4^+^ T cells from patients with active IBD. Microbiota-specific lamina propria T cell lines were obtained from intestinal specimens and exposed to *L. kefiri* CIDCA 8348 or conditioned medium to mitigate cell activation. The control of cell proliferation and cytokine secretion of activated T cells correlated significantly with previous results from our group found in healthy mice and in mice fed with a fructose-rich diet that were orally administered with this bacteria (P. Carasi et al., 2015; Zubiría et al., 2017).

Probiotic microorganisms isolated from kefir as well as some of their metabolites have been reported to reduce gut inflammation in colitis animal models, and the amelioration of symptoms after kefir consumption has been observed in one controlled trial with IBD patients to date (Iraporda et al., 2016; Sevencan et al., 2019; Yılmaz, Dolar, and Özpınar 2019). However, this is the first report to show the immunomodulation promoted by *Lactobacillus kefiri* in human organ culture *ex vivo*. The patients in our study were on immunosuppressive and/or immunomodulatory pharmacological treatment, and we considered this could be interfering with our *ex vivo* model results. However, mucosal biopsies obtained from inflamed colon areas of IBD patients showed increased basal levels of pro-inflammatory cytokines IL-6, IL-8 and even IL-1β and IL-17A, reflecting the periods of flares and increased disease activity occurring also in treated patients. We showed that *L. kefiri* CIDCA 8348 suppressed the secretion of these pro-inflammatory cytokines from IBD mucosal biopsies to similar levels as those found in healthy mucosa. Similar results were recently reported in a study performed by Pagnini et al., with a dose-response reduction of TNF-α and IL-17 expression in UC mucosal samples incubated with *L. rhamnosus* GG (Pagnini et al., 2018). In another study, IBD biopsies exposed to the probiotic *Lactococcus lactis* exhibited a reduced secretion of TNF-α and IL-23 (Simčič et al., 2019).

Considering that the gut barrier is impaired in IBD and luminal microorganisms may be found in the lamina propria of patients, we further addressed the effect of *L. kefiri* on lamina propria activated microbiota-specific T cells, to deeper understand whether *L. kefiri*’s anti-inflammatory effect occurred through lamina propria T lymphocyte modulation (Kumar et al., 2020; Al-Sadi et al., 2021). We isolated lamina propria mononuclear cells and established entero-adhesive *E. coli*-specific T cell lines for *in vitro* characterization. LPTC were activated with anti-CD3 and anti-CD28 antibodies, and we found that cell proliferation, cytokine secretion and NF-κB pathway activation were suppressed upon exposure to *L. kefiri*. Collectively, different strains of *Lactobacillus* and *Lactococcus* have shown to modulate the inflammatory activity through multiple mechanisms, but especially by inhibiting the translocation of the nuclear transcription factor NF-κB (Vincenzi et al.,. 2020; Zeng et al., 2020). Here, we co-incubated activated LPTC *in vitro* with *L. kefiri* and we found that NF-κB p65 was diminished in these cells, which also secreted lower amounts of TNF-α, IFN-γ, IL-6 and IL-13 than controls. In addition, activated LPTC incubated with *L. kefiri* CM also exhibited a reduced secretion of these cytokines. However, as proliferation of CD4^+^ T cells from these LPTC cultures was not diminished by *L. kefiri* CM, we propose that *L. kefiri* effect might be due to a direct interaction between the bacteria and the CD4^+^ T lymphocytes. To this regard, it has been previously described that the purified S-layer glycoprotein of *L. kefiri* CIDCA 8348 (a regular protein array that completely covers the bacterial surface) could enhance the activation of murine macrophages and bone marrow derived dendritic cells, through the recognition of the S-layer protein glycans by the C-type lectin receptor Mincle (Malamud et al., 2019). However, further investigations are needed to understand the role of the S-layer protein recognition on tuning the activation of antigen-presenting cells triggered by the whole bacteria. Nonetheless, a recent report by Gong et al. showed increased Mincle signaling in both intestinal samples from CD patients and experimental models of colitis, mainly due to an up-regulation of pyroptosis in macrophages, which promotes gut inflammation (Gong et al., 2020). However, the expression of Mincle on human CD4^+^ T lymphocytes has not been widely reported (Vijayan et al., 2010). Therefore the level of expression in LPTC from IBD patients and the possible role of this C-type lectin receptor in the interaction between LPTC CD4^+^ and *L. kefiri* CIDCA 8348 will be key issues to be addressed in further studies. The effect of conditioned medium on cytokine secretion may be attributed to metabolites secreted by lactobacilli. Of note, we have measured lactate present in *L. kefiri* conditioned medium used for the assay and it was 140 ± 0.2 µM. We have performed proliferation and cytokine assays including lactate as a possible modulator (Garrote et al., 2015; Iraporda et al., 2016), but results have been significant for concentrations above 10 mM (data not shown), a value much higher than that secreted by cultured *L. kefiri*. Hence the effect shown in this work cannot be attributed solely to this SCFA and must be further investigated.

Remarkably, in our study we found that LPTC incubated with *L. kefiri* or conditioned medium showed a high frequency of CD4^+^ FOXP3^+^ T cells. Several studies highlight that *Lactobacillus* may promote Treg differentiation in animal models (Zakostelska et al., 2011; Smelt et al., 2013; Park et al., 2017). Long term Treg cultures, like the ones performed in this work, have been shown to lose FOXP3 signaling upon repeated stimulation cycles (Hoffmann et al., 2009). In mice, *in vitro* incubation of CD4^+^ T cells with sonicated extracts of *Lactobacillus rhamnosus* GG diminished IL-17 secretion by these cells and increased FOXP3 and IL-10 secretion via TLR2 mechanism, clearly showing the plasticity induced by the probiotic (Jia et al., 2020). Our results could be showing the functional plasticity of human lamina propria effector T cells in response to the probiotic or its metabolites, which may induce FOXP3 induction. Our experiments showed a direct effect of *L. kefiri* on T cell IL-10 secretion, since no antigen presenting cells were included in our *in vitro* assays. Further studies are needed to characterize whether the FOXP3^+^ cells are responsible for the increase in IL-10 secretion, and functional assays should be performed to demonstrate the regulatory capacity of these CD4^+^ FOXP3^+^ T cells in the future. It is worth noting that the increase of IL-10 secretion induced by the probiotic may be important for generating a tolerogenic milieu in the inflamed gut, even in the absence of FOXP3 induction (Hoffmann et al., 2009). These effects, combined with a lower proliferation of effector CD4^+^ T lymphocytes and the decrease in pro-inflammatory cytokine secretion could be a useful complement of the adequate drug treatment to shift the IBD gut toward a tolerogenic state.

Although the anti-inflammatory activity of *L. kefiri* CIDCA 8348 has been shown in mice (Carasi et al., 2015; Zubiría et al., 2017), this is the first report demonstrating the immunomodulatory properties of a kefir-isolated strain (*L. kefiri* CIDCA 8348) on human intestinal tissue and primary T cells from IBD patients. Great effort has been made to modulate the pro-inflammatory activity of T cells as a treatment for IBD, with variable success; however, there is still need for improving therapies. Understanding the mechanisms of interaction between probiotics and immune cells in the gut could open new avenues to help prevent or treat inflammatory bowel disease.

## Data Availability

The original contributions presented in the study are included in the article/Supplementary Material, further inquiries can be directed to the corresponding author.
